# A Tea Plant (*Camellia sinensis*) *FLOWERING LOCUS C-like* Gene, *CsFLC1*, Is Correlated to Bud Dormancy and Triggers Early Flowering in *Arabidopsis*

**DOI:** 10.3390/ijms232415711

**Published:** 2022-12-11

**Authors:** Ying Liu, Ludovico Dreni, Haojie Zhang, Xinzhong Zhang, Nana Li, Kexin Zhang, Taimei Di, Lu Wang, Yajun Yang, Xinyuan Hao, Xinchao Wang

**Affiliations:** 1 Key Laboratory of Tea Biology and Resources Utilization, Ministry of Agriculture and Rural Affairs of the People’s Republic of China/ National Center for Tea Improvement/Tea Research Institute, Chinese Academy of Agricultural Sciences, Hangzhou 310008, China; 2 Instituto de Biología Molecular y Celular de Plantas (IBMCP), Consejo Superior de Investigaciones Científicas-Universidad Politécnica de Valencia, 46022 Valencia, Spain

**Keywords:** *FLC*, flowering, winter bud dormancy, seed germination, tea plant

## Abstract

Flowering and bud dormancy are crucial stages in the life cycle of perennial angiosperms in temperate climates. MADS-box family genes are involved in many plant growth and development processes. Here, we identified three *MADS-box* genes in tea plant belonging to the *FLOWERING LOCUS C (CsFLC)* family. We monitored *CsFLC1* transcription throughout the year and found that *CsFLC1* was expressed at a higher level during the winter bud dormancy and flowering phases. To clarify the function of *CsFLC1*, we developed transgenic *Arabidopsis thaliana* plants heterologously expressing *35S::CsFLC1*. These lines bolted and bloomed earlier than the WT (Col-0), and the seed germination rate was inversely proportional to the increased *CsFLC1* expression level. The RNA-seq of *35S::CsFLC1* transgenic *Arabidopsis* showed that many genes responding to ageing, flower development and leaf senescence were affected, and phytohormone-related pathways were especially enriched. According to the results of hormone content detection and RNA transcript level analysis, *CsFLC1* controls flowering time possibly by regulating *SOC1*, *AGL42*, *SEP3* and *AP3* and hormone signaling, accumulation and metabolism. This is the first time a study has identified *FLC-like* genes and characterized *CsFLC1* in tea plant. Our results suggest that *CsFLC1* might play dual roles in flowering and winter bud dormancy and provide new insight into the molecular mechanisms of *FLC* in tea plants as well as other plant species.

## 1. Introduction

Flowering is an important trait that helps plants transition from the vegetative phase to the reproductive phase and involves many complex changes, including those involving physiological, metabolic and molecular processes [1]. Moreover, flowering time is regulated not only by a plant’s intracellular signature but also by environmental factors [2]. Daylength and temperature are two main environmental factors that affect plant reproduction. According to the daylength and temperature, plants can sense the season and whether the time is appropriate to produce flowers and seeds. The vernalization pathway was proposed to explain how temperate angiosperms avoid blooming during winter. FLC is considered a crucial regulator in the vernalization pathway [3]. The function of *FLC* differs between ecotypes of *Arabidopsis thaliana*: it represses flowering in late-flowering ecotypes [4], while in early flowering ecotypes, *FLC* overexpression further delays flowering [5]. In Chinese cabbage (*Brassica rapa* ssp. *pekinensis* (Lour.) Hanelt), there are three *FLC* homologues that have lower expression levels in early flowering varieties [6]. Additionally, in *Eustoma grandiflorum* (Raf.) Shinners, *EgFLC* represses flowering [7]. FLC was reported to directly bind to the promoter of *SUPPRESSOR OF OVEREXPRESSION OF CONSTANS 1* (*SOC1*) and the first intron of *FLOWERING LOCUS T* (*FT*) to repress expression and delay flowering time [8,9]. *FLC* expression is reduced by DNA methylation (epigenetic modifications) but induced by acetylation of histones (chromatin remodeling) [10,11,12,13,14,15,16,17,18].

Bud dormancy is necessary for temperate perennials to avoid cellular damage and ensure plant survival during winter. *FLC* was also related to bud dormancy of perennial plant species such as apple (*Malus × domestica* Borkh.) and kiwifruit (*Actinidia chinensis* Planch.), where it is highly expressed during dormancy [19,20]. However, transgenic kiwifruit plants overexpressing *AcFLCL* displayed earlier budbreak times [20].

Tea plant (*Camellia sinensis* (L.) Kuntze) is an economically important crop species in many countries and areas of Asia, Africa and Latin America [21]. Its leaves are processed into different kinds of tea, which is famous for its health benefits [22,23,24,25]. Since tea plant is a crop species grown for its leaves, its reproductive phase from flowering to fruit production requires an abundance of plant resources, thus affecting tea plant bud growth and limiting the production and quality of tea. Therefore, breeding improved tea varieties or finding a way to balance vegetative and reproductive growth to improve the efficiency of the tea industry is urgently needed. For this, it is necessary to understand the molecular mechanisms of flowering in tea plant. Tea plant *FLC* orthologues have not yet been described. In our previous study, the phenotypes of two types of tea plant cultivars, a floriferous type and an oliganthous type, were observed [26]. We found that the meristem was maintained in the vegetative phase and did not switch to reproductive growth in the axillary buds of the oliganthous cultivars, while the transition to the floral meristem occurred in June in the axillary buds of the floriferous tea plant cultivars. The flowering-related genes in these two cultivars were identified, and among them, one MADS-box gene was found that might play an important role in floral organ differentiation and maturation [26]. In temperate areas, tea plant usually undergoes bud dormancy in the winter to overcome low-temperature stress, and when the temperature arises in the spring, the buds emerge. The progression of the bud dormancy–budbreak phase involves many genes, such as transcript factor genes bZIP (basic leucine-zipper) and MIKC-MADS as well as phytohormone-associated genes auxin-, ABA-, GA- and JA-associated genes [27,28,29]. Despite several studies about bud dormancy in tea plant, the role of *CsFLC* in dormancy is still unknown. Hence, it is necessary for breeders to understand the mechanisms of flowering and dormancy as well as their relationship.

Tea plant is a leaf-used crop, reproductive growth including flowering and fruit production affects vegetative growth and reduces outputting of tea. Therefore, it is necessary to study mechanisms of flowering in tea plant. In annuals, *FLC* plays key roles in flowering [3,4,5,6]. While in perennials, *FLC* is associated to bud dormancy [19,20]. Bud dormancy helps tea plant avoid low-temperature damage. *FLC-like* genes might have dual functions on flowering and bud dormancy in perennials. It’s meaningful to identify and characterize *FLC-like* genes in tea plant. In this study, we identified three *FLC-like* genes in tea plant, including *CsFLC1*, whose expression was correlated with bud dormancy and flower bud development. The RNA transcription of *CsFLC1* was monitored throughout the year. Then, we established *CsFLC1*-overexpressing transgenic *Arabidopsis* lines to characterize its functionality in a heterologous model plant species. Our study provides a new understanding of flowering as well as bud dormancy in tea plant and a theoretical basis for future breeding programmers to develop novel cultivars with early bud break and few flowers.

## 2. Results

### 2.1. Identification of CsFLC-Like Genes in Tea Genome

**Figure 1:**
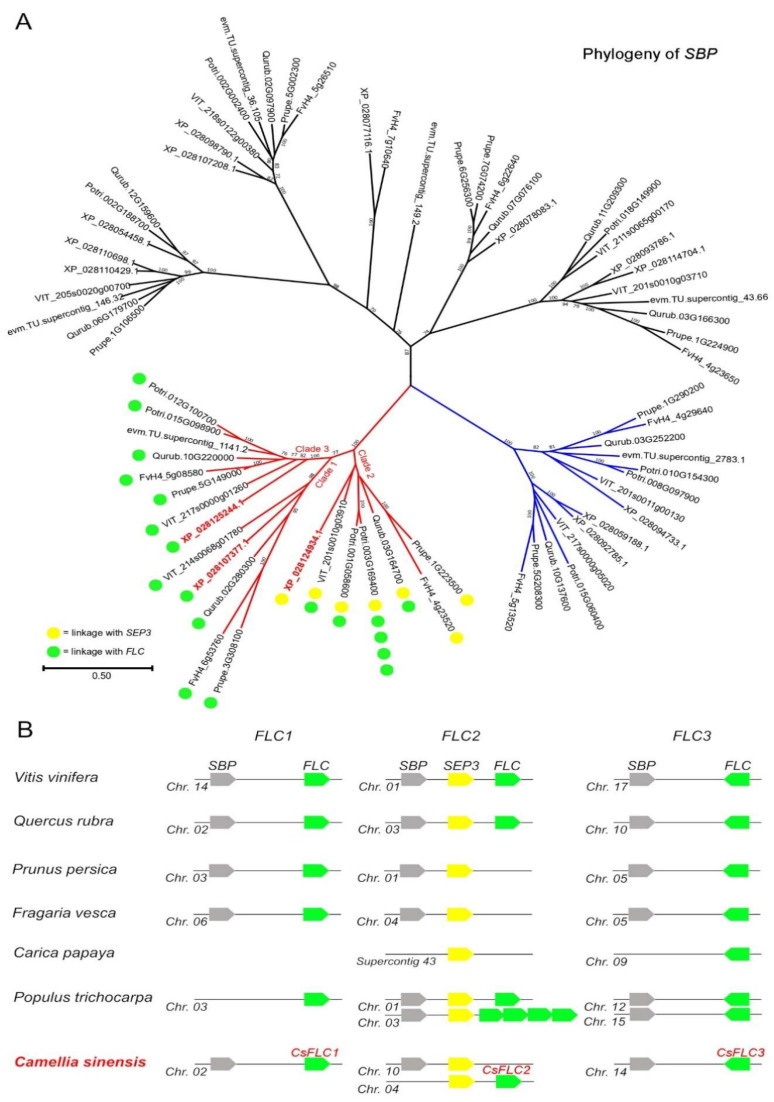
Identification and characterization of *CsFLC* genes. (**A**). As reported earlier by Ruelen et al. [30], *SBP* genes from a conserved monophyletic clade, shown in red, are closely linked to *SEP3* and *FLC*, and they are triplicated in core eudicots (clades 1, 2 and 3). Two of the three *SBP* genes that we found in *Camellia sinensis* show this conserved linkage with *FLC* genes. The genes of sister clade shown in blue are instead linked to *LOFSEP* and *SQUA* MADS-box genes. Potri: *Populus trichocarpa*; VIT: *Vitis vinifera*; XP: *Camellia sinensis*; Prupe: *Prunus persica*; FvH4: *Fragaria vesca*; Qurub: *Quercus rubra*; evm: *Carica papaya*. The tree was generated using protein sequences. Bootstrap values lower than 70 are not shown, and the scale bar indicates the number of amino acid sequence substitutions per site. (**B**). The three *SBP*-(*SEP3*)-*FLC* paralogous tandems of core eudicots show different patterns of conservation and duplications among species. In *Camellia sinensis*, which has an ancient tetraploid genome [32], six regions are expected; however, only duplicated copies of *SEP3* were retained. The results of this analysis suggest the presence of only three *FLC* genes in tea plant, which we refer to as *CsFLC1*, *CsFLC2* and *CsFLC3*.

In our previous work, we have isolated a MADS-box gene candidate that might play an important role in floral organ differentiation and maturation [26]. Then, we further analyzed its putative protein product and found that it shares the highest homology with *FUL*-like and *FLC*-like genes. Considering its high degree of sequence divergence, and in order to understand its true identity, we conducted a mixed approach of phylogeny and gene collinearity. A phylogenomic study [30] clarified that one paleo-*FLC* gene was present in the ancestor of angiosperms and that, in fact, *FLC*-like genes also exist in monocots but were previously misclassified. Their conserved genomic locations and their tendency to duplicate only by whole-genome duplications can help overcome the limits of phylogenetic analysis. In particular, the ancestral genomic configuration of *FLC* is in a narrow tandem with a monophyletic group of *SQUAMOSA promoter BINDING PROTEIN* (*SBP*) members and also with *SEPALLATA3* (*SEP3*) [30]. Since core eudicots belong to a paleohexaploid ancestor, this *SBP-SEP3-FLC* cluster should have triplicated in them. Indeed, in the model genome of grape (*Vitis vinifera* L.) two such clusters have been reported by Ruelen and coworkers [30] in addition to another one that we have found in chromosome 17 (Figure 1A,B). However, *FLC* and its close homologues *MAF* genes lost this conserved collinearity in the *Arabidopsis* family, the Brassicaceae, probably by gene transposition or a massive genome fractionation process [31].

Except for Brassicaceae, we found that the conservation of the three *SBP* paralogous clades is remarkable among core eudicot species, and most of them reveal an *FLC locus* in close proximity (Figure 1A,B). In tea plant, two of these *SBP* genes are located in proximity of a putative *FLC locus*, and an *SEP3-FLC* tandem also exists (Figure 1A,B). Despite the tea plant genome undergoing a relatively old whole-genome duplication [32]), only duplicated copies of *SEP3* seem to have been retained within the *SBP-SEP3-FLC* clusters, evidencing multiple gene losses (Figure 1B, Table S1). Finally, a fourth highly diverged MADS-box gene was isolated by BLAST (Genebank XP_028119527.1), without any clear homology to other core eudicot genes we have screened so far, and in a region non collinear to *FLC*, for which further analysis is needed. In conclusion, three paralogous *FLC* clades seem to exist in core eudicots, with one member each in the tea plant genome, that we named *CsFLC1*, *CsFLC2* and *CsFLC3*. Despite the fact that *CsFLC1* shares only 32% homology with Arabidopsis *FLC*, it is 56% homologous and collinear to the *AcFLCL* (Acc05562) gene recently reported in the closely related kiwifruit [20]. While *CsFLC2* is homologous and shares gene collinearity to kiwifruit genes Acc14299 and Acc33776 (data not shown).

Since *FLC-like* genes have higher expression during dormancy in apple and kiwifruit, we analyzed the expression pattern of *CsFLC1*, *CsFLC2* and *CsFLC3* in different tea bud dormant state transcriptomes [29], including endo-dormancy, eco-dormancy, para-dormancy and bud flush (Figure S1). The result shows that only *CsFLC1* had high mRNA expression level during winter dormancy (endo- and eco-dormancy) while *CsFLC2* did not have any change across the four states and *CsFLC3* had changes much lower than *CsFLC1*. Hence, *CsFLC1* was chosen for further analysis.

### 2.2. CsFLC1 Gene Expression Patterns

**Figure 2:**
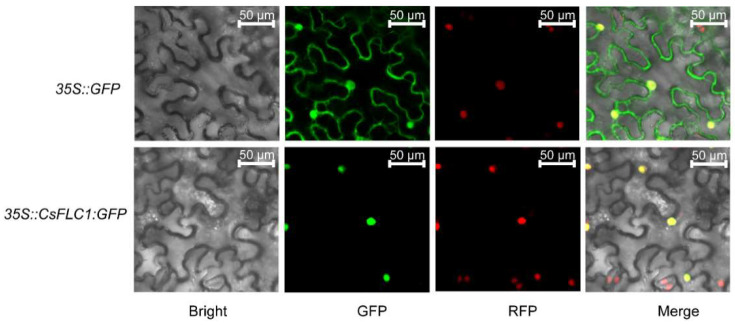
Subcellular location of CsFLC1 in *Nicotiana Benthamiana*. GFP: green fluorescent protein; RFP: red fluorescent protein; bar: 50 µm.

To identify the subcellular location of *CsFLC1*, we constructed a *35S::CsFLC1:eGFP* plasmid and transformed it into *Agrobacterium* to infect nuclear marker (red fluorescence) transgenic tobacco. Through confocal microscopy, we observed green fluorescence merged with red fluorescence to show yellow light. As expected for a putative transcription factor (TF), *CsFLC1* was located in the cell nucleus (Figure 2).

**Figure 3:**
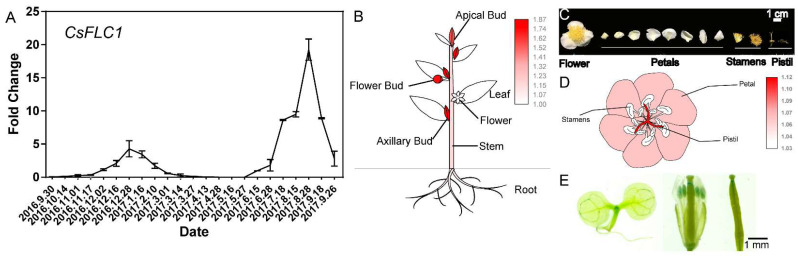
Expression patterns of *CsFLC1*. (**A**) Expression of *CsFLC1* in axillary or flower buds of tea plant throughout the year. (**B**) Different tissue expression of *CsFLC* in tea plant. (**C**) Various parts of flowers of tea plant. (**D**) Expression patterns of *CsFLC1* in different parts of flowers in tea plant. (**E**) GUS staining of *pCsFLC1::GUS* transgenic *Arabidopsis thaliana*.

To study the expression pattern of *CsFLC1*, we detected the gene expression level in axillary or floral buds in tea plant (Figure 3A). The result shows that *CsFLC1* had two expression peaks (one in 28 December and another in 28 August) in the whole year (from 2016 to 2017). From 1 November to the next 14 March was a dormancy period in tea plant [29]. From 27 May to 26 September was the floral bud differentiation and floral bud development phases [26]. The result shows that *CsFLC1* was corresponding to bud dormancy and flowering.

To identify in which tissues *CsFLC1* is expressed, we measured its expression in seven different tissues of tea plant, namely, apical buds, axillary buds, flower buds, flowers, mature leaves, stems and roots (Figure 3B). *CsFLC1* had the highest expression level in the three kinds of buds, and the next highest expression was in the stems and roots, while the lowest was in the flowers and leaves. Therefore, we further measured the expression in different flower organs (Figure 3C). The results show that *CsFLC1* had the highest expression level in the pistils followed by the petals, while it was not detected in stamens (Figure 3D). In *pCsFLC1::GUS* transgenic *Arabidopsis*, we found similar results (Figure 3E). *GUS* was expressed in the apical meristems, pistils, and stamens followed by the vascular tissue but was hardly detected in the roots and leaves. The expression patterns in tea and *pCsFLC1::GUS Arabidopsis* were common in the apical meristem and pistil but different in the petals, stamens and roots, which might be caused either by an incomplete *CsFLC1* promoter region or by differences in the *Arabidopsis* heterologous system.

In Figure 3A, *CsFLC1* was highly expressed during the bud dormancy phase, and to explore if *CsFLC1* could respond to low temperature (LT), we detected the expression pattern of *CsFLC1* under low-temperature treatment in tea plant.

**Figure 4:**
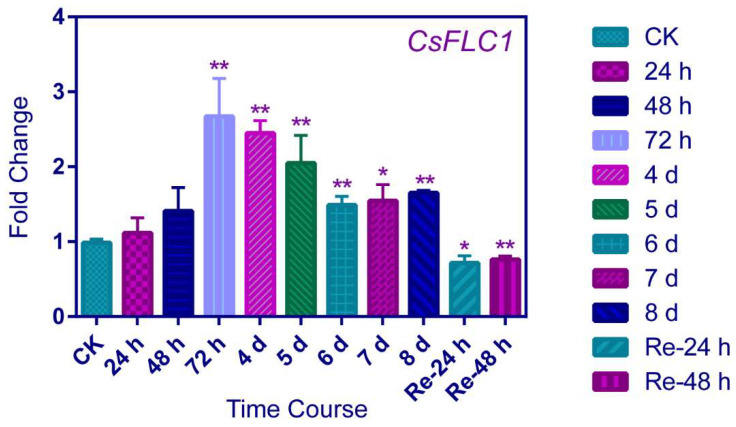
Expression pattern of *CsFLC1* under low-temperature treatment in tea plant. The *t*-test was applied for difference comparison between measurement data, and each was compared with CK; ** represented *p* < 0.01; * represented *p* < 0.05.

*CsFLC1* was significantly responding to LT after 72 h of treatment and then the RNA expression level was lower at 4, 5 and 6 days; later, at 7 and 8 days, the expression levels were stabilized but also higher than CK (0 d) (Figure 4). When recovering to a warm temperature after 24 and 48 h, the expression level of *CsFLC1* was significantly lower than CK. This result suggests that *CsFLC1* was induced by LT while being reduced by a warm temperature. Combining the expression pattern of *CsFLC1* in tea plant during bud dormancy, *CsFLC1* might play roles in maintaining bud dormancy.

### 2.3. The CsFLC1 Promoter Is Responsive to Low Temperature and Photoperiod

**Figure 5:**
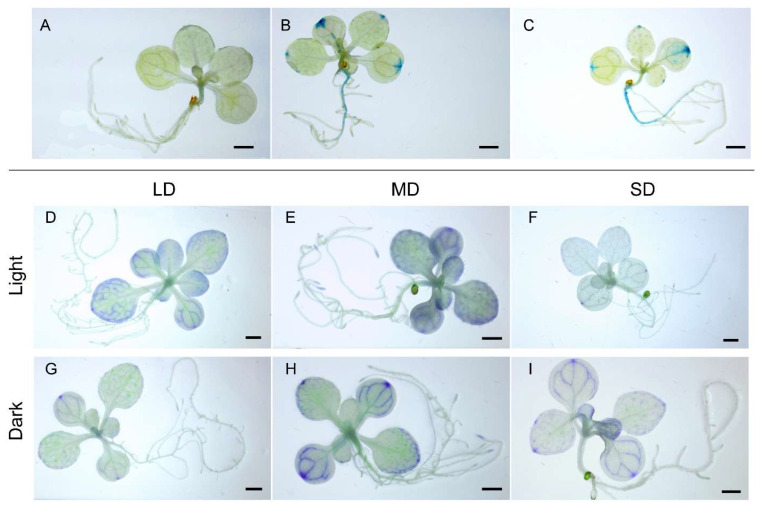
*CsFLC1* in response to low temperature and daylength. (**A**–**C**) *pCsFLC1::GUS* response to low temperature (LT; 4 °C): (**A**) before LT treatment; (**B**) after LT treatment for 3 h; (**C**) and after LT treatment for 4 h. (**D**–**F**) *pCsFLC1::GUS* staining in the light: (**D**) in plants under LDs; (**E**) in plants under MDs; and (**F**) in plants under SDs. (**G**–**I**) *pCsFLC1::GUS* staining in the dark: (**G**) in plants under LDs; (**H**) in plants under MDs; (**I**) and in plants under SDs. Bar = 1 cm; LD: long days; MD: medium days; SD: short days.

To clarify the *CsFLC1* response to environmental stimuli, we treated *pCsFLC1::GUS* transgenic lines with low temperature (4 °C) and different day length, LD (16 h light/8 h dark), medium day MD (12 h light/12 h dark) and short day SD (8 h light/16 h dark), and observed the relative GUS staining pattern. The result shows that the GUS staining in leaf veins, leaf apexes and roots was stronger under low temperature treatment than the control group (Figure 5A–C). GUS expressed highly either in light or dark under MD treatment (Figure 5E,H), while it expressed higher in light than dark under LD treatment (Figure 5D,G), and its behavior was the opposite under SD treatment (Figure 5F,I).

### 2.4. CsFLC1 Affects Flowering Time and Seed Germination in Transgenic Arabidopsis thaliana

**Figure 6:**
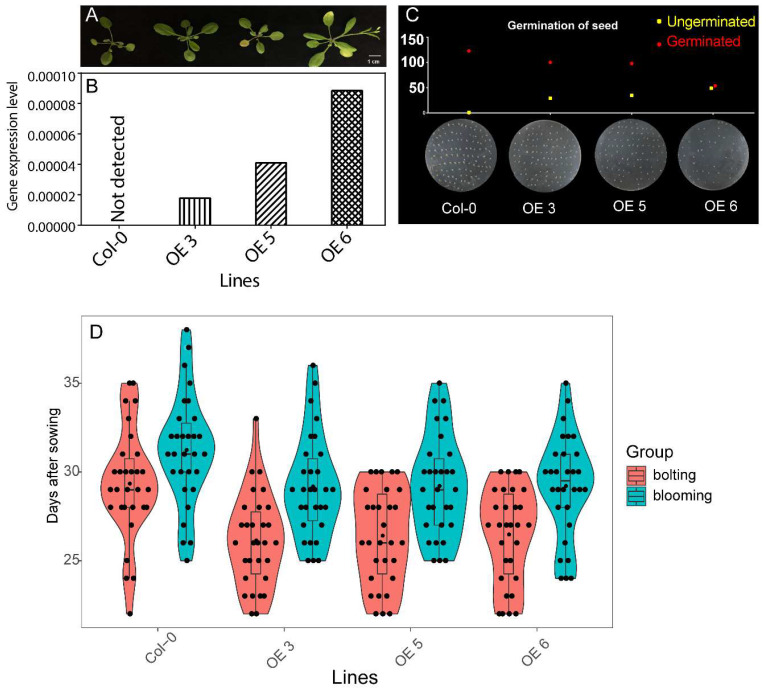
Phenotypes of *35S::CsFLC1* transgenic *Arabidopsis thaliana*. (**A**) Image of WT and *OE-CsFLC1* lines. (**B**) *CsFLC1* expression levels in different *Arabidopsis thaliana* lines. (**C**) Seed germination rates of different *Arabidopsis thaliana* lines. (**D**) Boxplot showing bolting and blooming times of different *Arabidopsis thaliana* lines: the bolting and blooming times were significant earlier in OE lines compared to WT, while there were no significant differences between OE lines.

To learn the function of *CsFLC1*, we tested three independent transgenic *Arabidopsis* lines of *35S::CsFLC1* (Figure 6B). We observed the phenotype of overexpressed (OE)lines and found the bolting, flowering and aging time of transgenic lines were earlier than Col-0 wild type (WT; Figure 6A,D). In addition, the higher the *CsFLC1* expression, the lower the seed germination rate (Figure 5C).

**Figure 7:**
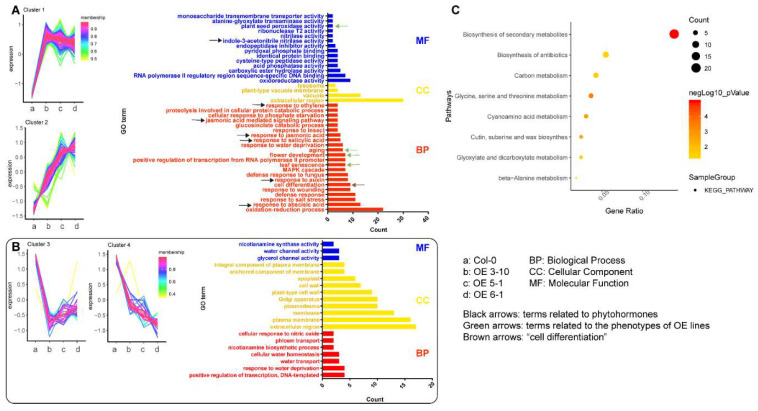
Transcriptome analysis of WT and *OE-CsFLC1* lines. (**A**) Gene cluster and GO enrichment of upregulated genes in *OE-CsFLC1* lines compared to WT. (**B**) Gene cluster and GO enrichment of downregulated genes in *OE-CsFLC1* lines compared to WT. (**C**) KEGG enrichment of upregulated genes in *OE-CsFLC1* lines compared to WT.

To further study how *CsFLC1* plays roles in these physiological processes, we detected gene expression levels of three OE lines and WT *Arabidopsis* by RNA-sequencing. Compared to WT, 228 differentially expressed genes (DEGs) were common in the three OE lines (Figure S2). In total, 169 DEGs were upregulated, while 59 DEGs were downregulated in OE lines compared with WT (Figure 7A,B). Among the up-regulated gene clusters of *OE-CsFLC1* lines, the terms ‘aging’, ‘flower development’ and ‘leaf senescence’ in biological process (BP) were enriched. Moreover, we observed that the *OE-CsFLC1* lines were senescent, bolting and blooming earlier than the wild type. Therefore, the phenotype and regulated pathways could be matched. The terms ‘response to abscisic acid’, ‘response to auxin’, ‘response to salicylic acid’, ‘response to jasmonic acid’, ‘jasmonic acid mediated signaling pathway’, ‘response to ethylene’ in BP and ‘indole-3-acetonitrile nitrilase activity’ in molecular function (MF) were enriched (Figure 7A). According to GO analysis in down-regulated gene clusters, the BP terms ‘water channel activity’, ‘glycerol channel activity’ and ‘nicotianamine synthase activity’, some cellular component (CC) terms about membrane and cell wall, as well as ‘cellular water homeostasis’, ‘water transport’ and ‘response to water deprivation’ MF terms were enriched (Figure 7B). The biosynthesis secondary metabolite KEGG pathway was clustered significantly in 169 upregulated DEGs, while no pathway was clustered in 59 downregulated DEGs (Figure 7C).

**Figure 8:**
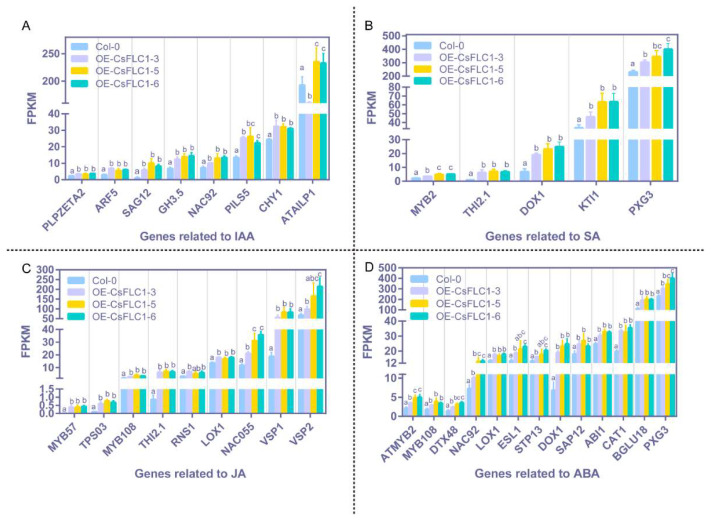
Expression of genes related to phytohormones in WT and *OE-CsFLC1* lines. (**A**) Expression of genes related to auxin in WT and *OE-CsFLC1* lines. (**B**) Expression of genes related to SA in WT and *OE-CsFLC1* lines. (**C**) Expression of genes related to JA in WT and *OE-CsFLC1* lines. (**D**) Expression of genes related to ABA in WT and *OE-CsFLC1* lines. IAA: indole-acetic acid; SA: salicylic acid; JA: jasmonic acid; ABA: abscisic acid. Different letters (a, b and c) on the column indicate significant difference between the data (*p* < 0.05).

To study which genes respond to auxin, SA, JA and abscisic acid (ABA) were upregulated in the high-level *CsFLC1* lines, and we displayed the transcriptional levels in the pathways mentioned above (Figure 8). The result shows that almost all these genes had a higher expression level in *OE-CsFLC1* lines compared to WT, but there were no significant differences of expression levels among the *OE-CsFLC1* lines. According to the results of transcriptomes, we noticed that three auxin-related genes *AUXIN RESPONSE FACTOR 5*/*MONOPTEROS* (*ARF5*/*MP*), *SENESCENCE-ASSOCIATED GENE 12* (*SAG12*) and the acyl acid amido synthetase gene *Gretchen Hagen 3.5* (*GH3.5*), three SA-related genes *THIONIN 2.1* (*THI2.1*), *MYB2* and *DIOXYGENASE 1* (*DOX1*), four JA-related genes *MYB57*, *TERPENE SYNTHASE* 03 (*TPS03*), *NAC055* and *VEGETATIVE STORAGE PROTEIN 1* (*VSP1*) as well as four ABA-related genes *DETOXIFICATION 48* (*DTX48*), *NAC92*, *CATALASE 1* (*CAT1*) and *BETA GLUCOSIDASE 18* (*BGLU18*) were significantly and obviously upregulated in *OE-CsFLC1* lines.

**Figure 9:**
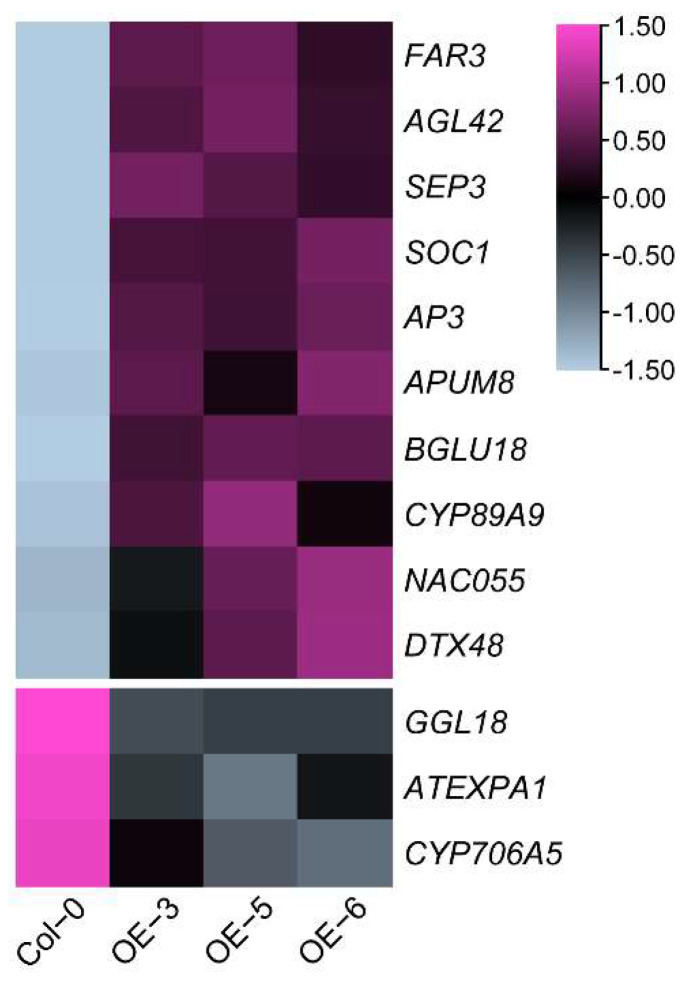
Expression patterns of *Arabidopsis* AtFLC target genes [33] in WT and *OE-CsFLC1* transgenic *Arabidopsis thaliana*. The pink color represents upregulated genes, the blue color represents downregulated genes, and the heatmap was generated by TBtools.

To explore which DEGs might be regulated by *CsFLC1* and cause the phenotypes of transgenic *Arabidopsis thaliana*, we analyzed expression patterns of known *AtFLC* direct target genes based on ChIP-seq data [33]. There were 13 common genes, among them 10 genes were induced, while 3 genes were repressed in *OE-CsFLC1* lines (Table S2). In *OE-CsFLC1* lines, four MADS-box family members *AGAMOUS-LIKE 42* (*AGL42*), *SEP3*, *SOC1* and *APETALA3* (*AP3*), three hormone-related genes *BGLU18*, *NAC055* and *DTX48*, a post-transcriptional regulator *PUMILIO 8* (*APUM8*), a Cytochrome P450 gene *CYP89A9* as well as a wax biosynthetic gene *FATTY ACID REDUCTASE 3* (*FAR3*) were up-regulated while *GUARD-CELL-ENRICHED GDSL LIPASE 18* (*GGL8*), a cell-wall-related gene *EXPA1* as well as a Cytochrome P450 gene *CYP706A5* were down-regulated (Figure 9).

In conclusion, *CsFLC1* has functions in controlling bolting and blooming times, seed germination, leaf senescence as well as regulating some auxin, SA-, JA- and ABA-associated genes in transgenic *Arabidopsis thaliana*.

### 2.5. Phytohormone Contents of Tea Plants in the Whole Year

**Figure 10:**
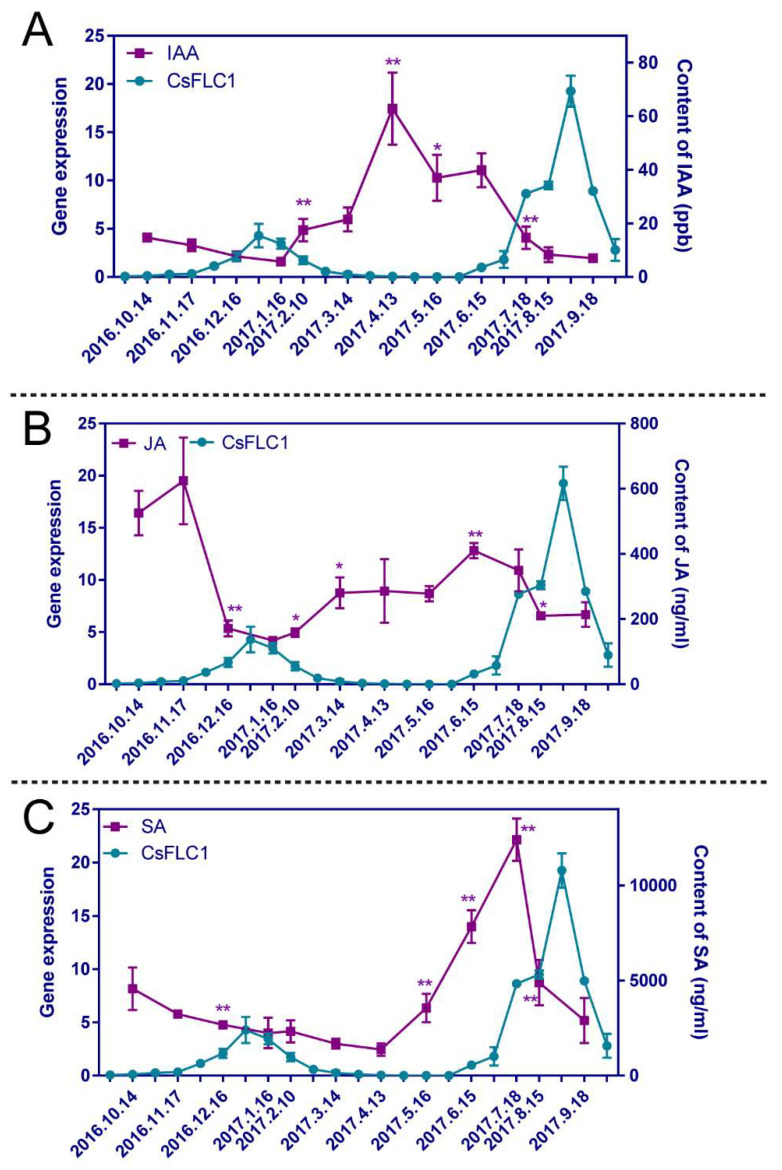
Variation trends of different phytohormone contents in tea plant during the whole year. (**A**) Content of IAA in tea plant throughout the year. (**B**) JA content of tea plant throughout the year. (**C**) Content of SA in tea plant throughout the year. The t-test was applied for difference comparison between measurement data, and each was compared with the previous one; ** represented *p* < 0.01; * represented *p* < 0.05. IAA: indole-3-acetic acid; JA: jasmonic acid; SA: salicylic acid.

Since we found there were some phytohormone pathways influenced in *35S::CsFLC1* lines, we measured the contents of three hormones (indole-acetic acid (IAA), JA and SA) in tea plant buds throughout the year. The hormone content changes are displayed in Figure 10.

The content of IAA was low from 14 October 2016 to 16 January 2017, and then increased until 13 April. The content peaked; later, it declined until 18 July, and finally, it was stable and low again (Figure 10). The JA content was high on 14 October and 17 November, decreased on 16 December, remained at a low level until 10 February, increased on 14 March, remained at a moderate level from 10 February to 16 May, increased on 15 June, decreased until 15 August, and finally remained low until 18 September (Figure 10B). The content of SA was 4573.43 ng/mL on 14 October 2016, slowly decreased until 13 April 2017, rapidly increased until 18 July and rapidly decreased until 18 September, sharply peaking on 18 July (Figure 10C). The changes in IAA and JA contents were opposite those of the *CsFLC1* expression level. There was only one time at which the SA content peak, which occurred one and-a-half months before the peak *CsFLC1* expression occurred, which was on 28 August. In conclusion, *CsFLC1* expression coincided with low contents of IAA and JA throughout the year but coincided with a high SA content of during flowering.

## 3. Discussion

### 3.1. Function of CsFLC in Reproduction Processes

Flowering time is regulated by five main pathways: the autonomous, gibberellin, aging, photoperiod and vernalization pathways [34,35]. These various pathways depended on different genetic regulations [36]. *FLC* was identified first in 1999 and was considered to encode a repressor of flowering; its expression was upregulated by *FRIGIDA* (*FRI*) but downregulated by vernalization in Landsberg erecta (Ler, a late-flowering ecotype) [4,10]. The *FLC* expression level was found to be an indicator of the extent of the vernalization and was also regulated by the autonomous pathway [3,37]. *FLC* regulates the circadian clock via autonomous and vernalization pathways to control flowering time, which shows that *FLC* is a link between vernalization and the circadian rhythm [38]. In addition, by binding to their chromatin, FLC represses the expression of *FT* and *SOC1* to inhibit flowering [8,9]. In apple trees, *MdFLC-like* is expressed in flower buds and is upregulated during cold accumulation and flower primordium differentiation and development [19].

In our previous study, we found that, during the flowering period, *CsFLC1* was specifically expressed during the floral transition stage [26]. In this study, to find out when *CsFLC1* was functioning, we detected the expression pattern of *CsFLC1* in tea plant. According to the expression patterns, *CsFLC1* was highly expressed during flower organ development. Thus, it positively regulated floral development, which coincided with the function of the apple *MdFLC-like* gene. Based on these findings, we constructed transgenic *CsFLC1* OE *Arabidopsis* in the Col-0 (an early flowering ecotype) background to further study the function of *CsFLC1*. With respect to *35S::CsFLC1* transgenic lines, bolting and blooming occurred earlier than it did for Col-0. To clarify which pathways and genes were influenced by *CsFLC1* in OE *Arabidopsis*, we performed RNA-seq. According to the GO analysis of the transcriptome data, genes annotated to the ‘flower development’ term were enriched in the OE lines. We measured *CsFLC1* expression in the apical and axillary buds, flower buds and pistils of tea plant as well as in the carpel of *pCsFLC1::GUS* transgenic *Arabidopsis* to research the tissue expression of *CsFLC1*. Our results show that *CsFLC1* might positively regulate early flowering, floral transition, petal and pistil development reproduction processes. The transcriptome results show that *SOC1* was upregulated in *OE-CsFLC1* lines. Thus, *CsFLC1* might induce flowering by influencing the expression of *SOC1* in tea plant, the results of which are the opposite to those of Ler *Arabidopsis* plants for both phenotype and genetic regulatory relationships. *AGL42*, a *SOC1*-*like* gene that was reported to be a target of *SOC1* and *FLC*, can promote flowering [33,39]. *SEP3* is essential for floral meristem determinacy [40]. *AP3 is* involved in the formation of petals and stamens during flower development [41]. In our study, these four MADS-box genes *SOC1*, *AGL42*, *SEP3* and *AP3* were upregulated by *CsFLC1*, indicating that *CsFLC1* promotes flowering by controlling the expression of these genes in *Arabidopsis*. While in *flc-3* mutant, AtFLC targets *SOC1* and *SEP3* were up-regulated [33], it was the same in *OE-CsFLC1* transgenic *Arabidopsis* (Figure 9), which means that AtFLC and CsFLC1 have the opposite function on regulating *SOC1* and *SEP3*. The fact that *CsFLC1* promotes flowering while *AtFLC* represses flowering might be because the sequences of *FLC* are not conserved among species [30], which results in different functions in flowering and gene regulation.

### 3.2. CsFLC1 Putative Function in Bud Dormancy

In tea plant, *CsFLC1* was highly expressed during the winter, which was identified as the endodormancy period of axillary buds (Figure S1). Based on the results of the GUS location patterns, *CsFLC1* was highly expressed in light under LDs, while it was induced in the dark under SDs, but it was highly expressed continuously under MDs. Interestingly, *CsFLC* accumulated in the day under LD conditions but accumulated at night under SD conditions (Figure 3). This means that it plays different roles under different photoperiods. In previous studies, the transcript of *FLC* was independent of the photoperiod [3,4], indicating that *CsFLC1* is a special *FLC* with dual function in response to different photoperiods. Additionally, in the *pCsFLC1::GUS* lines as well as LT-treated tea plant (Figure 4 and Figure 5), *CsFLC1* could respond to low temperatures. SDs and low temperatures are the signals of winter, and plants enter dormancy when they receive the signals of winter to avoid damage and no longer grow before the environment is suitable for growth.

To further explore whether *CsFLC1* was coordinated with bud dormancy, we detected its expression in the late budbreak cultivar ZHDB from 30 September 2016 to 16 May 2017 (Figure S3). The results show that the mRNA transcript of *CsFLC1* was no longer detected approximately one month later than in LJ43, which coincided with budbreak time. Combining the results above, we predict that CsFLC1 is a repressor of bud break and maintains the bud dormancy state. In apple and kiwifruit, *FLC-like* genes were reported to be highly expressed during dormancy, while overexpression *AcFLCL* transgenic kiwifruit budbreak time is earlier [19,20]. This might be because *FLC* plays a role like a switch on bud dormancy only if *FLC* reaches a threshold value when the environmental temperature is favorable, meaning the dormancy could break easily. In tea plant, *CsFLC1* was up-regulated from endo- to eco-dormancy (Figure 3A and Figure S1); in addition, *CsFLC1* could be a response to low temperatures and down-regulated by the following warm temperature (Figure 4), which indicates that *CsFLC1* might be a switch between bud dormancy and budbreak. Phytohormones play important roles in regulating plant bud dormancy [42]. In tea plant, auxin-, ABA-, GA- and JA-associated genes were involved in bud dormancy [29,43]. In *OE-CsFLC1* transgenic *Arabidopsis*, IAA-, JA- and ABA-associated genes were affected (Figure 7A and Figure 8), which suggested that *CsFLC1* might control bud dormancy by regulating phytohormone-associated genes.

### 3.3. Relationship between CsFLC1 Expression and Phytohormones’ Concentration and Response

Phytohormones are important in the growth, development and response to environmental stimuli of plants. It has been reported that flowering and growth are promoted by relatively low concentrations of auxin but inhibited by higher concentrations [44]. According to the GO enrichment of DEGs of *OE-CsFLC1 Arabidopsis*, several phytohormones, such as auxin, SA and JA were enriched (Figure 7). To clarify the relationship between *CsFLC1* and hormones, contents of IAA, SA and JA were detected. The results of whole-year RNA expression and hormone content detection showed that *CsFLC1* RNA transcription was correlated with a low content of IAA in both axillary and floral buds of tea plant. ARFs are TFs that bind to AuxREs in the promoters of early auxin response genes [45,46]. *ARF5* is an important activator in auxin signaling and is expressed in cells with low levels of auxin [47]. *SAG12* is a senescence indicator and is repressed by auxin [48,49,50]. *GH3.5* functions in modulating and integrating both auxin and SA signaling [51,52] is expressed in seedlings, roots, stems, buds and blooming flowers of *Arabidopsis thaliana* [53], and influences root development in rice [54]. *ARF5*, *SAG12* and *GH3.5* were significantly upregulated in *OE-CsFLC1* lines, which indicated that *CsFLC1* might activate them to control auxin signaling and further induce flowering at low concentrations of auxin.

JA plays a critical role in inflorescence, stamen and seed development [55,56]. JA induces the expression of *MYB57* to promote stamen filament development [57]. The JA content moderately peaked from 16 May to 15 August when the plants were in the floral transition and floral organ differentiation stages, and *MYB57* had an obviously higher transcription level in *OE-CsFLC1* lines. *NAC055* is a target of FLC and is induced by MeJA [58]; in our study, it was highly expressed in *OE-CsFLC1* transgenic *Arabidopsis*. These results show that both *CsFLC1* and JA might control flowering through *NAC055*.

Cleland and Ajami [59] found that SA could induce flowering in *Lemna gibba* G3. In this study, the content of SA increased during floral induction and initiation stages in tea plant and then decreased after 18 July. The results suggest that SA may play roles in the floral induction of tea plant, which is in accordance with the results of previous studies on *Arabidopsis thaliana* [60]. *NAC055* could reduce the accumulation of SA [61], and it was upregulated by *CsFLC1*. Therefore, the subsequent decrease in SA content might be because the high expression of *CsFLC1* induces the expression of *NAC055*. *DOX1* is expressed in the roots, anthers, and senescing leaves and induced by SA [62]. According to the results, the above *DOX1* expression level was increased in the *OE-CsFLC1* lines, which indicated that flowering induction and leaf senescence by *CsFLC1* and SA might occur through the *DOX1*-dependent signaling pathway.

ABA inhibits seed germination while accelerating floral transition and flower development [63,64,65,66]. ABA application can enhance *CAT1* expression in maize embryos [67]. Based on the transcriptome results, *CAT1* expression was also significantly increased in the *OE-CsFLC1* lines. *MYB2* is a repressor of proanthocyanidins, and anthocyanin biosynthesis also plays a positive role in seed dormancy [68]. Therefore, we proposed that *OE-CsFLC1* lines have defective seed germination, possibly because of the upregulation of ABA signaling genes, including *CAT1* and *MYB2*.

The contents of IAA, JA and SA in tea plant, as well as the RNA expression level of hormone-related genes in *OE-CsFLC1* transgenic *Arabidopsis*, which indicates that *CsFLC1* might play roles in flowering through these hormones’ signaling, accumulation and metabolism, have not been reported in other species until now, even though there were some FLC targets associated to phytohormones that existed in *Arabidopsis thaliana* [33].

In conclusion, *CsFLC1* was found to be involved in flowering in *Arabidopsis thaliana* and tea plant, possibly by influencing flowering-related genes (*SOC1*, *AGL42*, *SEP3* and *AP3*) and hormone signaling, accumulation and metabolism.

## 4. Materials and Methods

### 4.1. Plant Materials and Growth Conditions

Buds, leaves, flowers, stems and roots of Longjing 43 (LJ43) and buds of Zhenghedabai (ZHDB) tea plant cultivars for gene expression or hormone detection were collected from a tea plantation at the Tea Research Institute of the Chinese Academy of Agricultural Sciences (Hangzhou, China; N30°180′, E120°100′). Axillary buds located at the same positions along the branches from more than 30 individual plants were collected in the afternoon on 30 September, 14 October, 1 November, and 16 December 2016, and on 16 January, 10 February, 1 March, 14 March, 27 March, 13 April, 28 April, 16 May, 27 May, 15 June, 28 June and 18 July 2017. Flower buds were collected in the afternoon on 15 August, 28 August, 18 September and 26 September 2017. The apical, axillary and floral buds as well as the leaves, flowers, stems and roots of LJ43 were collected on 12 April 2019, for tissue expression analysis. There were three biological replicates collected at each time point. All *Arabidopsis thaliana* plants except those composing the treatment groups were grown under long days (LDs; 16 h light/8 h darkness) at 22 °C and under 70% relative humidity. As for low-temperature treatment tea plant (LJ43), potted plants were grown under LDs (14 h light/10 h darkness) at 25 °C and under 75% relative humidity for 7 d to naturalize in climatic chamber. Then, the tea plant was treated at 4 °C for 8 d, and the temperature recovered to 25 °C to treat the tea plant for 2 d. One bud and three leaves were collected for RNA extraction. There were three biological replicates collected at each time point (4 °C 0 d (CK), 24 h, 48 h, 72 h, 4 d, 5 d, 6 d, 7 d, 8 d as well as recover 24 and 48 h). Tissues of *Arabidopsis thaliana* and tea plant for RNA extraction were frozen in liquid nitrogen immediately after harvesting and stored at −80 °C before use.

### 4.2. Phylogenetic Analysis

The two tea plant genome assemblies, namely, GCA_004153795.1 [69] and GCA_013676235.1 [70], available in the NCBI database were screened to identify the *FLC* genes and their collinear genes. Only the latter assembly was used to study conserved gene collinearity because of its high continuity at the chromosome-scale level. The other genes and genomes were accessed through Phytozome v.13; several incomplete annotations were found that we corrected by screening the NCBI GenBank database. Gene collinearity was assessed by SynFind (https://genomevolution.org/coge/SynFind.pl, accessed on 11 July 2022) and manually.

To construct a phylogenetic tree, SBP proteins were aligned using MAFFT (https://mafft.cbrc.jp/alignment/server/, accessed on 11 July 2022) and then analyzed with MEGA 11 [71]. The evolutionary history was inferred by using the maximum likelihood method and the Jones Taylor Thornton (JTT) matrix-based model. The trees were drawn to scale, with branch lengths equal to the number of substitutions per site. The accessions used are listed in Figure 1 and in Supplementary Table S1.

### 4.3. Quantitative Real-Time PCR (qRT-PCR) Analysis

Total RNA was isolated from tissues of tea plant as well as *Arabidopsis thaliana* using an RNAprep Pure Plant Kit (Tiangen Biotech Co., Ltd., Beijing, China) according to the manufacturer’s protocols. For qRT-PCR, 1 μg of total RNA was used to synthesize first-strand cDNA with PrimeScript RT enzyme together with gDNA eraser (Takara, Kyoto, Japan). qRT-PCR was performed on a Roche LightCycler 480 (Roche Diagnostics, Rotkreuz, Switzerland) using LightCycler 480 SYBR Green I Master Reagent (Roche Diagnostics, Rotkreuz, Switzerland). The gene-specific primer pairs used are listed in Table S3. The polypyrimidine tract-binding protein (*CsPTB1*) gene was used as an internal control [72]. The relative expression levels were calculated using the 2^−ΔΔCt^ method [73].

### 4.4. Cloning, Plasmid Construction and Transformation

Due to the current lack of efficient tea plant transformation techniques, we used the model plant species *Arabidopsis thaliana* as a heterologous expression system to validate *CsFLC1′s* function. The primers that we designed to clone the promoter and coding DNA sequence (CDS) of *CsFLC1* are listed in Table S3. The CDS of *CsFLC1* was cloned into pCAMBIA1300 (p1300) together with a 35S promoter vector to obtain the *35S::CsFLC1* p1300 plasmid. The 1066 bp region upstream of the ATG codon of *CsFLC1* was cloned into p1300 together with a β-glucuronidase (*GUS*) fragment vector, yielding *pCsFLC1::GUS* p1300 plasmids. We transformed the plasmids into *Agrobacterium tumefaciens* GV3101. Transgenic *35S::CsFLC1* and *pCsFLC1::GUS Arabidopsis thaliana* plants were obtained by the floral dip technique [74]. The resulting T_0_ generation seeds were germinated on 15 mg/L hygromycin B selective 1/2-strength Murashige and Skoog (MS) plates to select positive plants from among those composing the T_1_ generation, and then the positive plants were transplanted into soil to collect T_1_ seeds. The T_1_ seeds were sown on selective MS plates as described above to select T_2_-generation seedlings showing a positive:negative selection ratio ≈ 3:1; these seedlings were transplanted into soil to obtain T_2_-generation seeds. The T_2_ seeds were plated on selective MS plates as described above to select the lines that were 100% hygromycin-resistant, which constituted T_3_-generation homozygous lines.

### 4.5. Subcellular Localization

To obtain *35S::CsFLC1:eGFP* recombination plasmids, we cloned the CDS of *CsFLC1* into p1300 together with the 35S promoter and a *GFP* reporter vector. Then, we transformed the resulting plasmids into *Agrobacterium tumefaciens* GV3101. Transformation of *Nicotiana benthamiana* with nuclear marker was performed according to a previous description [75]. An Olympus FV1000 confocal laser-scanning microscope (Zeiss, Oberkochen, Germany) was used for imaging.

### 4.6. GUS Staining

For cold treatment, seven-day-old *pCsFLC1::GUS Arabidopsis* transgenic lines were exposed to 4 °C for 0, 3 or 4 h. For photoperiod treatment, *pCsFLC1::GUS* transgenic seedlings were grown under 3 different daylengths: LDs (16 h light/8 h darkness), medium days (MDs; 12 h light/12 h darkness) and short days (SDs; 8 h light/16 h darkness) after germinating for one week. Seedlings were collected at the end of the light or dark phase. Different samples or tissues of transgenic *Arabidopsis thaliana pCsFLC1::GUS* plants were subjected to GUS staining buffer as previously described [76]. Two independent T_3_ homozygous lines were used for GUS staining in each experiment. An Olympus SZ61 microscope and a Nikon Eclipse 80i microscope were used for imaging.

### 4.7. RNA Sequencing and Transcriptome Data Analysis of Arabidopsis Transgenic Lines

Leaves from twenty-two-day-old Arabidopsis Col-0 and three different *CsFLC1* overexpression T3 homozygous lines (OE 3-10, OE 5-1 and OE 6-1) were collected to perform RNA sequencing. Twelve plants were pooled as one biological replicate, and three replicates of each line and of ecotype Columbia (Col-0) were used for RNA sequencing. A Venn diagram was generated by TBtools [77]. Gene expression pattern clusters were evaluated via R version 3.6.1. Gene Ontology (GO) enrichment was analyzed by GOEAST tools [78]. Kyoto Encyclopedia of Genes and Genomes (KEGG) functional annotation clustering was performed by using DAVID [79].

### 4.8. Measurements of Phytohormone Contents in Tea Plant Buds

For hormone extraction and content determination, 0.1 g of buds of tea plant that were the same samples as those used for RNA extraction were ground into powder in liquid nitrogen; there were three biological replicates for each time point. The methods that we used to extract and determine the contents of the phytohormones have been described previously [80]. Diagrams of the results were generated by GraphPad Prism 6 software (USA).

## Supplementary Materials

The following supporting information can be downloaded at: https://www.mdpi.com/article/10.3390/ijms232415711/s1.

## Abbreviations

ABA	Abscisic acid
AP3	APETALA3
ARF5/MP	AUXIN RESPOSE FACTOR 5/MONOPTEROS
CAT1	CATALASE 1
CAL	CAULIFLOWER
CDS	Coding sequences
DEGs	Differentially expressed genes
DOX1	DIOXYGENASE 1
FT	FLOWERING LOCUS T
FLC	FLOWERING LOCUS C
GO	Gene ontology
GH3.5	Gretchen Hagen 3.5
IAA	Indoleacetic acid
JA	Jasmonic acid
KEGG	Kyoto Encyclopedia of Genes and Genomes
LD	Long day
MD	Middle day
MF	Molecular function
OE	Overexpression
SA	Salicylic acid
SAG12	SENESCENCE-ASSOCIATED GENE 12
SHP	SHATTERPROOF
SAMs	Shoot apical meristems
SD	Short day
SOC1	SUPPRESSOR OF OVEREXPRESSION OF CO 1
TPS03	TERPENE SYNTHASE 03
THI2.1	THIONIN 2.1
TF	Transcription factor
VSP1	VEGETATIVE STORAGE PROTEIN 1

## References

[1] Balanzà: V., Martinez-Fernandez I., Sato S., Yanofsky M.F., Kaufmann K., Angenent G.C., Bemer M., Ferrandiz C. (2018). Genetic control of meristem arrest and life span in *Arabidopsis* by a FRUITFULL-APETALA2 pathway. Nat. Commun..

[2] Dong X., Li Y., Guan Y., Wang S., Luo H., Li X., Li H., Zhang Z. (2021). Auxin-induced AUXIN RESPONSE FACTOR4 activates *APETALA1* and *FRUITFULL* to promote flowering in woodland strawberry. Hort. Res..

[3] Sheldon C.C., Rouse D.T., Finnegan E.J., Peacock W.J., Dennis E.S. (2000). The molecular basis of vernalization: The central role of *FLOWERING LOCUS C* (*FLC*). Proc. Natl. Acad. Sci. USA..

[4] Michaels S.D., Amasino R.M. (1999). *FLOWERING LOCUS C* encodes a novel MADS domain protein that acts as a repressor of flowering. Plant Cell.

[5] Ratcliffe O.J. (2001). Regulation of flowering in *Arabidopsis* by an *FLC* homologue. Plant Physiol..

[6] Kim S.Y., Park B.S., Kwon S.J., Kim J., Lim M.H., Park Y.D., Kim D.Y., Suh S.C., Jin Y.M., Ji H.A. (2007). Delayed flowering time in *Arabidopsis* and *Brassica rapa* by the overexpression of *FLOWERING LOCUS C* (*FLC*) homologs isolated from Chinese cabbage (*Brassica rapa* L. ssp. *pekinensis*). Plant Cell Rep..

[7] Nakano Y., Kawashima H., Kinoshita T., Yoshikawa H., Hisamatsu T. (2011). Characterization of *FLC*, *SOC1* and *FT* homologs in *Eustoma grandiflorum*: Effects of vernalization and post-vernalization conditions on flowering and gene expression. Physiol. Plant..

[8] Searle I. (2006). The transcription factor FLC confers a flowering response to vernalization by repressing meristem competence and systemic signaling in *Arabidopsis*. Genes Dev..

[9] Seo E., Lee H., Jin J., Park H., Kim J., Noh Y.S., Lee I. (2009). Crosstalk between cold response and flowering in *Arabidopsis* is mediated through the flowering-time gene *SOC1* and its upstream negative regulator FLC. Plant Cell.

[10] Sheldon C.C., Burn J.E., Perez P.P., Metzger J., Dennis E.S. (1999). The *FLF* MADS Box gene: A repressor of flowering in *Arabidopsis* regulated by vernalization and methylation. Plant Cell.

[11] Questa J.I., Song J., Geraldo N., An H., Dean C. (2016). *Arabidopsis* transcriptional repressor VAL1 triggers Polycomb silencing at *FLC* during vernalization. Science.

[12] He Y., Amasino R.M. (2005). Role of chromatin modification in flowering-time control. Trends Plant Sci..

[13] Greb T., Mylne J.S., Crevillen P., Geraldo N., An H., Gendall A.R., Dean C. (2007). The PHD finger protein VRN5 functions in the epigenetic silencing of *Arabidopsis FLC*. Curr. Biol..

[14] Sheldon C.C., Hills M.J., Lister C., Dean C., Dennis E.S., Peacock W.J. (2008). Resetting of *FLOWERING LOCUS C* expression after epigenetic repression by vernalization. Proc. Natl. Acad. Sci. USA..

[15] Xiao J., Zhang H., Xing L., Xu S., Liu H., Chong K., Xu Y. (2013). Requirement of histone acetyltransferases HAM1 and HAM2 for epigenetic modification of *FLC* in regulating flowering in *Arabidopsis*. Plant Physiol..

[16] Kwak J.S., Son G.H., Song J.T., Seo H.S. (2016). Post-translational modifications of *FLOWERING LOCUS C* modulate its activity. J. Exp. Bot..

[17] Kwak J.S., Son G.H., Sung-Il K., Song J.T., Seo H.S. (2016). *Arabidopsis* HIGH PLOIDY2 sumoylates and stabilizes *FLOWERING LOCUS C* through its E3 ligase activity. Front. Plant Sci..

[18] Nishio H., Buzas D.M., Nagano A.J., Suzuki Y., Sugano S., Ito M., Morinaga S.I., Kudoh H. (2016). From the laboratory to the field: Assaying histone methylation at *FLOWERING LOCUS C* in naturally growing *Arabidopsis halleri*. Genes Genet. Syst..

[19] Soichiro N., Cecile M.M., Hisayo Y., Chikako H., Kazuma O., Yosuke T., Shigeki M., Tao R. (2019). Functional and expressional analyses of apple *FLC-like* in relation to dormancy progress and flower bud development. Tree Physiol..

[20] Voogd C., Brian L.A., Wu R., Wang T., Allan A.C., Varkonyi-Gasic E. (2022). A MADS-box gene with similarity to *FLC* is induced by cold and correlated with epigenetic changes to control budbreak in kiwifruit. N. Phytol..

[21] Wang X., Feng H., Chang Y., Ma C., Wang L., Hao X., Li A., Cheng H., Wang L., Cui P. (2020). Population sequencing enhances understanding of tea plant evolution. Nat. Commun..

[22] Malar D.S., Prasanth M.I., Brimson J.M., Sharika R., Sivamaruthi B.S., Chaiyasut C., Tencomnao T. (2020). Neuroprotective properties of green tea (*Camellia sinensis*) in Parkinson’s Disease: A Review. Molecules.

[23] Yi M., Wu X., Zhuang W., Xia L., Chen Y., Zhao R., Wan Q., Du L., Zhou Y. (2019). Tea consumption and health outcomes: Umbrella review of meta-analyses of observational studies in humans. Mol. Nutr. Food Res..

[24] Xu X.Y., Zhao C.N., Cao S.Y., Tang G.Y., Gan R.Y., Li H.B. (2020). Effects and mechanisms of tea for the prevention and management of cancers: An updated review. Crit. Rev. Food Sci. Nutr..

[25] Williams J.L., Everett J.M., D’Cunha N.M., Sergi D., Georgousopoulou E.N., Keegan R.J., McKune A.J., Mellor D.D., Anstice N., Naumovski N. (2019). The effects of green tea amino acid L-theanine consumption on the ability to manage stress and anxiety levels: A systematic review. Plant Foods Hum. Nutr..

[26] Liu Y., Hao X., Lu Q., Zhang W., Zhang H., Wang L., Yang Y., Xiao B., Wang X. (2020). Genome-wide identification and expression analysis of flowering-related genes reveal putative floral induction and differentiation mechanisms in tea plant (*Camellia sinensis*). Genomics.

[27] Wang X.C., Hao X.Y., Ma C.L., Cao H.L., Yue C., Wang L., Zeng J.M., Yang Y.J. (2014). Identification of differential gene expression profiles between winter dormant and sprouting axillary buds in tea plant (*Camellia sinensis*) by suppression subtractive hybridization. Tree Genet. Genomes.

[28] Chen Y., Li Y., Ren H., Zhou J., Wang L., Yang Y., Hao X., Wang X. (2021). Genome-wide identification and expression profiling reveal the diverse role of Methyl-CpG-binding domain proteins in tea plant (*Camellia sinensis*). Beverage Plant Res..

[29] Hao X., Yang Y., Yue C., Wang L., Horvath D.P., Wang X. (2017). Comprehensive transcriptome analyses reveal differential gene expression profiles of *Camellia sinensis* axillary buds at para-, endo-, ecodormancy, and bud flush stages. Front. Plant Sci..

[30] Ruelens P., Maagd R.A.D., Proost S., Theien G., Geuten K., Kaufmann K. (2013). *FLOWERING LOCUS C* in monocots and the tandem origin of angiosperm-specific MADS-box genes. Nat. Commun..

[31] Zhao T., Holmer R., Bruijn S.D., Angenent G.C., Van D., Schranz M.E. (2017). Phylogenomic synteny network analysis of MADS-Box transcription factor genes reveals lineage-specific transpositions, ancient tandem duplications, and deep positional conservation. Plant Cell.

[32] Chen J.D., Zheng C., Ma J.Q., Jiang C.K., Ercisli S., Yao M.J., Chen L. (2020). The chromosome-scale genome reveals the evolution and diversification after the recent tetraploidization event in tea plant. Hortic. Res..

[33] Deng W., Ying H., Helliwell C.A., Taylor J.M., Peacock W.J., Dennis E.S. (2011). *FLOWERING LOCUS C* (*FLC*) regulates development pathways throughout the life cycle of *Arabidopsis*. Proc. Natl. Acad. Sci. USA..

[34] Amasino R.M., Michaels S.D. (2010). The timing of flowering. Plant Physiol..

[35] He Y., Chen T., Zeng X. (2020). Genetic and epigenetic understanding of the seasonal timing of flowering. Plant Commun..

[36] Bloomer R.H., Dean C. (2017). Fine-tuning timing: Natural variation informs the mechanistic basis of the switch to flowering in *Arabidopsis thaliana*. J. Exp. Bot..

[37] Michaels S.D. (2001). Loss of *FLOWERING LOCUS C* activity eliminates the late-flowering phenotype of FRIGIDA and autonomous pathway mutations but not responsiveness to vernalization. Plant Cell.

[38] Salathia N., Davis S.J., Lynn J.R., Michaels S.D., Millar A.J. (2006). *FLOWERING LOCUS C*-dependent and -independent regulation of the circadian clock by the autonomous and vernalization pathways. BMC Plant Biol..

[39] Dorca-Fornell C., Gregis V., Grandi V., Coupland G., Colombo L., Kater M.M. (2011). The *Arabidopsis SOC1-like* genes *AGL42*, *AGL71* and *AGL72* promote flowering in the shoot apical and axillary meristems. Plant J..

[40] Véronique H., Silva C.S., Agnès J., Arnaud S., Quentin C., Vanessa C., Conn S.J., Carles C.C., François P., Chloe Z. (2018). Tetramerization of MADS family transcription factors *SEPALLATA3* and *AGAMOUS* is required for floral meristem determinacy in *Arabidopsis*. Nucleic Acids Res..

[41] Wuest S.E., O"Maoileidigh D.S., Rae L., Kwasniewska K., Raganelli A., Hanczaryk K., Lohan A.J., Loftus B., Graciet E., Wellmer F. (2012). Molecular basis for the specification of floral organs by *APETALA3* and *PISTILLATA*. Proc. Natl. Acad. Sci. USA..

[42] Ito A., Tuan P.A., Saito T., Bai S., Kita M., Moriguchi T. (2021). Changes in phytohormone content and associated gene expression throughout the stages of pear (*Pyrus pyrifolia* Nakai) dormancy. Tree Physiol..

[43] Hao X., Tang H., Wang B., Wang L., Cao H., Wang Y., Zeng J., Fang S., Chu J., Yang Y. (2019). Gene characterization and expression analysis reveal the importance of auxin signaling in bud dormancy regulation in tea plant. J. Plant Growth Regul..

[44] Leopold A., Thimann K. (1949). The effect of auxin on flower initiation. Am. J. Bot..

[45] Guilfoyle T.J., Hagen G. (2007). Auxin response factors. Curr. Opin. Plant Biol..

[46] Tiwari S.B., Hagen G., Guilfoyle T. (2003). The roles of auxin response factor domains in auxin-responsive transcription. Plant Cell.

[47] Cucinotta M., Cavalleri A., Guazzotti A., Astori C., Manrique S., Bombarely A., Oliveto S., Biffo S., Weijers D., Kater M.M. (2021). Alternative splicing generates a *MONOPTEROS* isoform required for ovule development. Curr. Biol..

[48] Noh Y.S., Amasino R.M. (1999). Identification of a promoter region responsible for the senescence-specific expression of *SAG12*. Plant Mol. Biol..

[49] Grbić V. (2003). SAG2 and SAG12 protein expression in senescing *Arabidopsis* plants. Physiol. Plant..

[50] James M., Poret M., Masclaux-Daubresse C., Marmagne A., Coquet L., Jouenne T., Chan P., Trouverie J., Etienne P. (2018). SAG12, a major cysteine protease involved in nitrogen allocation during senescence for seed production in *Arabidopsis Thaliana*. Plant Cell Physiol..

[51] Staswick P.E., Tiryaki I., Rowe M.L. (2002). Jasmonate response locus *JAR1* and several related *Arabidopsis* genes encode enzymes of the firefly luciferase superfamily that show activity on jasmonic, salicylic, and indole-3-acetic acids in an assay for adenylation. Plant Cell.

[52] Zhang Z., Li Q., Li Z., Staswick P.E., Wang M., Zhu Y., He Z. (2007). Dual regulation role of *GH3. 5* in salicylic acid and auxin signaling during *Arabidopsis*-*Pseudomonas syringae* interaction. Plant Physiol..

[53] Zhang Z., Wang M., Li Z., Li Q., He Z. (2008). *Arabidopsis GH3. 5* regulates salicylic acid-dependent and both *NPR1*-dependent and independent defense responses. Plant Signal. Behav..

[54] Fu J., Yu H., Li X., Xiao J., Wang S. (2011). Rice GH3 gene family: Regulators of growth and development. Plant Signal. Behav..

[55] Wasternack C., Forner S., Strnad M., Hause B. (2013). Jasmonates in flower and seed development. Biochimie.

[56] Yuan Z., Zhang D. (2015). Roles of jasmonate signalling in plant inflorescence and flower development. Curr. Opin. Plant Biol..

[57] Cheng H., Song S., Xiao L., Soo H.M., Cheng Z., Xie D., Peng J. (2009). Gibberellin acts through jasmonate to control the expression of *MYB21*, *MYB24*, and *MYB57* to promote stamen filament growth in *Arabidopsis*. PLoS Genet..

[58] Hickman R., Van Verk M.C., Van Dijken A., Mendes M.P., Vroegop-Vos I.A., Caarls L., Steenbergen M., Van der Nagel I., Wesselink G.J., Jironkin A. (2017). Architecture and dynamics of the jasmonic acid gene regulatory network. Plant Cell.

[59] Cleland C.F., Ajami A. (1974). Identification of the flower-inducing factor isolated from aphid honeydew as being salicylic acid. Plant Physiol..

[60] Martínez C., Pons E., Prats G., León J. (2004). Salicylic acid regulates flowering time and links defence responses and reproductive development. Plant J..

[61] Zheng X.Y., Spivey N.W., Zeng W., Liu P.P., Fu Z.Q., Klessig D.F., He S.Y., Dong X. (2012). Coronatine promotes Pseudomonas syringae virulence in plants by activating a signaling cascade that inhibits salicylic acid accumulation. Cell Host Microbe.

[62] De León I.P., Sanz A., Hamberg M., Castresana C. (2002). Involvement of the *Arabidopsis* α-DOX1 fatty acid dioxygenase in protection against oxidative stress and cell death. Plant J..

[63] Giraudat J. (1995). Abscisic acid signaling. Curr. Opin. Cell Biol..

[64] Hubbard K.E., Nishimura N., Hitomi K., Getzoff E.D., Schroeder J.I. (2010). Early abscisic acid signal transduction mechanisms: Newly discovered components and newly emerging questions. Genes Dev..

[65] Fitzpatrick A.H., Shrestha N., Bhandari J., Crowell D.N. (2011). Roles for farnesol and ABA in Arabidopsis flower development. Plant Signal. Behav..

[66] Conti L., Galbiati M., Tonelli C., Zhang D.P. (2014). ABA and the floral transition. Abscisic acid: Metabolism, transport and signaling.

[67] Guan L.M., Zhao J., Scandalios J.G. (2000). Cis-elements and trans-factors that regulate expression of the maize *Cat1* antioxidant gene in response to ABA and osmotic stress: H_2_O_2_ is the likely intermediary signaling molecule for the response. Plant, J..

[68] Jun J.H., Liu C., Xiao X., Dixon R.A. (2015). The transcriptional repressor MYB2 regulates both spatial and temporal patterns of proanthocyandin and anthocyanin pigmentation in *Medicago truncatula*. Plant Cell.

[69] Wei C., Yang H., Wang S., Zhao J., Liu C., Gao L., Xia E., Lu Y., Tai Y., She G. (2018). Draft genome sequence of *Camellia sinensis* var. *sinensis* provides insights into the evolution of the tea genome and tea quality. Proc. Natl. Acad. Sci. USA..

[70] Zhang W., Zhang Y., Qiu H., Guo Y., Wan H., Zhang X., Scossa F., Alseekh S., Zhang Q., Wang P. (2020). Genome assembly of wild tea tree DASZ reveals pedigree and selection history of tea varieties. Nat. Commun..

[71] Tamura K., Stecher G., Kumar S. (2021). MEGA11: Molecular evolutionary genetics analysis version 11. Mol. Biol. Evol..

[72] Hao X., Horvath D.P., Chao W.S., Yang Y., Wang X., Xiao B. (2014). Identification and evaluation of reliable reference genes for quantitative real-time PCR analysis in tea plant (*Camellia sinensis* (L.) O. Kuntze). Int. J. Mol. Sci..

[73] Schmittgen T.D., Livak K.J. (2008). Analyzing real-time PCR data by the comparative C(T) method. Nat. Protoc..

[74] Bent A. (2006). *Arabidopsis thaliana* floral dip transformation method. Methods Mol. Biol..

[75] Kapila J., De Rycke R., Van Montagu M., Angenon G. (1997). An *Agrobacterium*-mediated transient gene expression system for intact leaves. Plant Sci..

[76] Wang L., Yao L., Hao X., Li N., Qian W., Yue C., Ding C., Zeng J., Yang Y., Wang X. (2018). Tea plant SWEET transporters: Expression profiling, sugar transport, and the involvement of *CsSWEET16* in modifying cold tolerance in *Arabidopsis*. Plant Mol. Biol..

[77] Chen C., Chen H., Zhang Y., Thomas H.R., Frank M.H., He Y., Xia R. (2020). TBtools: An integrative toolkit developed for interactive analyses of big biological data. Mol. Plant.

[78] Zheng Q., Wang X.J. (2008). GOEAST: A web-based software toolkit for Gene Ontology enrichment analysis. Nucleic Acids Res..

[79] Dennis G., Sherman B.T., Hosack D.A., Yang J., Gao W., Lane H.C., Lempicki R.A. (2003). DAVID: Database for annotation, visualization, and integrated discovery. Genome Biol..

[80] Lu Q., Wang Y., Xiong F., Hao X., Zhang X., Li N., Wang L., Zeng J., Yang Y., Wang X. (2020). Integrated transcriptomic and metabolomic analyses reveal the effects of callose deposition and multihormone signal transduction pathways on the tea plant-*Colletotrichum camelliae* interaction. Sci. Rep..

